# A systematic review of newborn health interventions in humanitarian settings

**DOI:** 10.1136/bmjgh-2022-009082

**Published:** 2022-07-01

**Authors:** Mariana Rodo, Diane Duclos, Jocelyn DeJong, Chaza Akik, Neha S Singh

**Affiliations:** 1 Health in Humanitarian Crises Centre, London School of Hygiene & Tropical Medicine, London, UK; 2 Department of Epidemiology and Population Health, Faculty of Health Sciences, American University of Beirut, Beirut, Lebanon; 3 Center for Research on Population and Health, Faculty of Health Sciences, American University of Beirut, Beirut, Lebanon

**Keywords:** systematic review, child health, health services research, health policy

## Abstract

**Background:**

Almost half of the under-5 deaths occur in the neonatal period and most can be prevented with quality newborn care. The already vulnerable state of newborns is exacerbated in humanitarian settings. This review aims to assess the current evidence of the interventions being provided in these contexts, identify strategies that increase their utilisation and their effects on health outcomes in order to inform involved actors in the field and to guide future research.

**Methods:**

Searched for peer-reviewed and grey literature in four databases and in relevant websites, for published studies between 1990 and 15 November 2021. Search terms were related to newborns, humanitarian settings, low-income and middle-income countries and newborn health interventions. Quality assessment using critical appraisal tools appropriate to the study design was conducted. Data were extracted and analysed using a narrative synthesis approach.

**Results:**

A total of 35 articles were included in this review, 33 peer-reviewed and 2 grey literature publications. The essential newborn care (ENC) interventions reported varied across the studies and only three used the Newborn Health in Humanitarian Settings: Field Guide as a guideline document. The ENC interventions most commonly reported were thermal care and feeding support whereas delaying of cord clamping and administration of vitamin K were the least. Training of healthcare workers was the most frequent strategy reported to increase utilisation. Community interventions, financial incentives and the provision of supplies and equipment were also reported.

**Conclusion:**

There is insufficient evidence documenting the reality of newborn care in humanitarian settings in low-income and middle-income countries. There is a need to improve the reporting of these interventions, including when there are gaps in service provision. More evidence is needed on the strategies used to increase their utilisation and the effect on health outcomes.

**PROSPERO registration number:**

CRD42020199639.

**WHAT IS ALREADY KNOWN ON THIS TOPIC**

- Newborn health is particularly susceptible to socioeconomic environment, and therefore to conflict and other crises in humanitarian settings where in turn health services are often fragmented and access to healthcare weaker.
- About half of the global under-5 mortality happens in the neonatal period and humanitarian crisis-affected settings carry an increased burden of these deaths; but there has been no systematic review to date focused on newborn health interventions in humanitarian settings.

**WHAT THIS STUDY ADDS**

- The types of interventions of newborn care reported vary across studies—thermal care and feeding support are the most commonly reported while delaying cord clamping and administration of vitamin K the least.
- Training of health workers, community health interventions, financial incentives and provision of specialised resources are reported strategies to increase utilisation.
- Most studies do not assess interventions’ effect on newborn health outcomes.

**HOW THIS STUDY MIGHT AFFECT RESEARCH, PRACTICE OR POLICY**

- More research is needed that specifically focuses on newborn care provided in communities and health facilities, and the strategies used to increase and improve them; and there is a need to standardise and increase reporting of newborn health interventions both by implementers and researchers.

## Introduction

The number of people living in humanitarian situations is increasing. It is estimated that 274 million people will need humanitarian assistance and protection in 2022.^1^ The overall disruption of health services and reduced access to healthcare contribute to increasing the state of vulnerability and poor health outcomes for people living in these situations, resulting in an excess morbidity and mortality.^2–4^ In these critical situations, women and children under 5 years of age are disproportionately affected.^5^

Despite the rapid improvement in child survival in the last two decades, progress is uneven across age groups.^6^ In 2017, it was reported that 47% of the global under-5 mortality occurred in the neonatal period and that crisis-affected countries are the ones carrying the highest burden of these deaths.^6 7^ Of the six countries with the highest neonatal mortality rate in 2018, five of them were affected by humanitarian crises.^6^

Newborns are defined as children aged 0–28 days after birth. They are, by nature, at risk of high mortality and their survival is sensitive to their socioeconomic environment.^8^ Many of these deaths are preventable with appropriate quality care that can be delivered in low-resource settings including contexts of humanitarian crises.^7^

Newborn care cannot be provided in isolation and should be accompanied by maternal care. This is because newborns are part of a continuum of care that have historically been included in sexual and reproductive health (SRH) services, from prepregnancy to birth and the postnatal period.^7 9^ Despite its natural connection with maternal and child health in the continuum of care, until the mid-2000s newborn health was largely overlooked within these health areas.

SRH needs of populations in humanitarian settings were first prioritised in the mid-1990s, driven by the increase in recognition of the needs of refugees and displaced populations. This took form with the creation of the Inter-Agency Working Group (IAWG) on Reproductive Health in Refugee Situations.^10^ This led to the development of the Inter-Agency Field Manual (IAFM), which includes guidelines to provide reproductive health services, first specifically for refugee settings and later in 2010, expanded for broader humanitarian settings.^10^ This Manual outlines the Minimum Initial Service Package (MISP), a minimum set of health interventions that should be provided from the initial phase of a humanitarian crisis, and also covers comprehensive services to be included when the situation stabilises.^10^

In 2014, WHO’s global action plan ‘Every Newborn’ raised awareness on preventable neonatal deaths and set up goals to decrease these numbers by 2035.^11^ In 2018, the Newborn Health in Humanitarian Settings: Field Guide (subsequently referred to as ‘Field Guide’) was developed to complement the existing IAFM with more detailed guidance concerning newborn health in humanitarian settings.^7^

The set of newborn health interventions identified in the Field Guide starts from pregnancy to labour/childbirth and newborn care, in the immediate and late postnatal period.^7^ Given that the time around labour and birth accounts for the highest proportion maternal and newborn deaths globally,^12^ intervention packages that focus on this period are the ones with the highest potential to reduce preventable newborn deaths.^11^ The Field Guide suggests a range of interventions across the different levels of care, which can be implemented around this period. These are in line with the intervention packages set out by WHO’s Global Action Plan and are also included in the roadmap as some of the key actions to accelerate the progress of newborn health.^13^ Specific for the postnatal period, the mentioned newborn interventions are essential newborn care (ENC)—a set of interventions that should be provided to all newborns, regardless of the place of birth^7^—and care of small and sick newborns^7 11^ (table 1).

**Table 1:** Newborn health interventions as per Field Guide^7^

Essential newborn care (ENC)	
Initiation of breathing*	Drying, clearing airway, stimulation through rubbing the back and neonatal resuscitation (using bag and mask), if required.
Thermal care	Drying, warming, skin-to-skin and delayed bathing of a newborn.
Hygiene and infection prevention	Clean birth practices, hand washing and clean cord/skin/eye care. Use of clean and dry cord care or use chlorhexidine according to the setting’s neonatal mortality.
Feeding support	Skin-to-skin contact, support for exclusive and immediate breast feeding and not discarding colostrums.
Monitoring of danger signs	Frequent assessment for danger signs of serious infections and other conditions that require more specialised care.
Postnatal care checks	Visits in the first month of life (24 hours, 3 days and 7–15 days).
Delay of cord clamping and administration of vitamin K†	
Care of small and sick newborns	
Extrathermal care, including Kangaroo Mother Care when possible. Additional support for feeding of small and preterm newborns. Treatment of infections and jaundice.† Adequate use of oxygen therapy.	

*This set of interventions is included in the MISP but not as part of the ENC. These interventions are part of the EmONC and neonatal resuscitation is one of the signal functions.

†Not included in the MISP.

MISP, Minimum Initial Service Package.

Even with these guiding documents, a recent review looked at the availability of global guidance to care for women, newborns, children and adolescents in conflict settings and reported a gap in guidance related to newborn health.^14^

To date, no systematic review has been conducted on newborn health interventions in humanitarian settings. A study in 2017 that systematically reviewed the evidence on public health interventions in humanitarian settings suggested a gap in the evidence based on newborn health.^15^ From the previous systematic reviews focusing on the effectiveness, utilisation of and evaluation of SRH services and programmes,^2 16 17^ only one reported a study where ENC was evaluated.^2^ A 2017 review assessed the use of services of the dyad maternal-newborn health in fragile and conflict-affected areas in Asia and the Middle East, although it did not focus exclusively on humanitarian settings or on newborn health.^18^

In light of the increased emphasis on improving coverage and quality of newborn health interventions, this study aims to provide a comprehensive synthesis of the existing evidence on newborn health interventions that are being provided in humanitarian settings, the strategies employed to increase their utilisation and their effect on health outcomes. The purpose of the research is to inform involved actors in this field and to guide future research.

## Methods

This systematic review follows the Preferred Reporting Items for Systematic Reviews and Meta-Analyses statement.^19^ It is registered in PROSPERO database with the identifier number CRD42020199639. The full inclusion and exclusion criteria are presented in table 2.

**Table 2:** Inclusion and exclusion criteria for literature in the systematic review

	Included	Excluded
Population of interest	Newborns (0–28 days after birth)^7^ living in humanitarian settings in low-income and middle-income countries.	Populations living in humanitarian setting in high-income countries.
Intervention	Any intervention directly aimed at improving newborn health outcomes as defined by the MISP and the Field Guide as per table 1.	Any intervention not included in the MISP and Field Guide, prepartum and intrapartum interventions and other sexual and reproductive health interventions not impacting newborn health.
Outcome	Studies reporting on the use or effectiveness of interventions that affect newborn health.	Studies that do not report on the use or effectiveness of newborn health interventions.
Situation	Studies conducted during acute or protracted humanitarian crises (armed conflict, disease outbreak, natural disaster).	Studies conducted before or after the crisis has fully stabilised (ie, no longer a protracted crisis).
Type of study	Quantitative, qualitative and mixed methods studies describing a newborn health intervention, including or not an evaluation of an outcome, and reporting empirical data.	Any other types of studies not reporting empirical data, for example, comments, opinion pieces.
Type of publication	Peer-reviewed and grey literature.	Other types of publications, for example, media articles, blogs.
Publication date	1 January 1990–15 November 2021.	Any publication published before 1 January 1990.
Language	English, Portuguese, Spanish, French.	Other languages.

MISP, Minimum Initial Service Package.

A humanitarian setting is defined by IAWG^10^ as the result of one or a set of events that pose a critical threat to the health, safety and well-being of a community and can be manmade, have natural causes or combine several of these causes. Because the majority of crises occur in low-income and middle-income countries (LMICs) and high-income countries (HICs) typically have the resources to manage such situations,^2^ for the purpose of this review, only LMICs are included.

Although interventions during pregnancy and birth also impact newborn health outcomes, for this review, we focused on the ones directly related to newborns as per guidance from the MISP and the Field Guide^7 10^ (table 1).

### Search strategy

The search strategy was based on previous systematic reviews on SRH in humanitarian settings.^2 17^ The search terms used fit the categories of newborn, newborn health and its specific interventions, humanitarian settings and LMICs. Free text and subject headings were used and adapted to each database. The full search strategy is included in online supplemental file 1.

10.1136/bmjgh-2022-009082.supp1 Supplementary data

The peer-reviewed literature search was done using the following databases: Medline, Embase, Global Health and PsycINFO from 1 January 1990 to 15 November 2021 as previous reviews found no articles on newborn health before this date.

The grey literature search was conducted on the following platforms: IAWG, Save the Children, Médecins Sans Frontières (MSF), International Rescue Committee, Jphiego, CARE International, International Committee of the Red Cross, RAISE Initiative, United Nations Population Fund, UNICEF, Healthy Newborn Network, Women’s Refugee Commission (WRC), Results for Development, Marie Stopes International and Population Council. General terms such as ‘newborn/neonatal health’, ‘humanitarian’ were used for these searches. Advanced searches using similar terms were run on Google Scholar. Reference lists of relevant systematic reviews, including peer-reviewed papers and grey literature publications, were screened for additional articles.

### Study selection and data extraction

Results from database and grey literature searches were exported to EndNote X9. MR and NSS independently conducted double screening, with titles and abstracts screened for relevance, and then full-text articles screened against inclusion criteria (table 2). Grey literature articles were screened first by title and then by full text for possible inclusion.

Data were extracted from each included study by MR using Microsoft Excel, and independently checked by NSS for both accuracy and completeness. Data were extracted on the following domains: author and year, study setting, target population, crisis type, study design, intervention domain, intervention description, results and implementing actors.

### Analysis

For the analysis of the results, a narrative synthesis was conducted as it best suits the diversity of studies found, and has been used in previous reviews of SRH in humanitarian settings.^17 20 21^ The synthesis of results was done by categorising the type of intervention reported, identifying any strategies used to increase utilisation and effectiveness and the implementers of these interventions.

The quality of each included article was critically appraised using the appropriate tools for the type of study reported. Interventional studies were appraised using the Consolidated Standards of Reporting Trials^22^; observational studies were appraised using the Strengthening of Observational Studies in Epidemiology checklist^23^; the National Heart, Lung and Blood Institute’s checklist for pre-post studies with no control group^24^; the Mixed Methods Appraisal Tool for mixed methods studies and controlled pre-post studies^25^; the Critical Appraisal Skills Programme checklist was used for qualitative studies^26^ and the Centre for Evidence-Based Medicine checklist for case studies.^27^

All the included articles were given a final score, which was converted to a percentage. Based on this final percentage, the articles were given a rating of low, medium or high quality. Low-quality studies scored between 0% and 33%, medium-quality studies between 34% and 66% and those scoring >67% were considered high-quality studies. This quality threshold was used in this review as it has been used in previous reviews of SRH interventions in humanitarian settings.^2 17^

### Patient and public involvement

Patients and public were not directly involved in this review; we used publicly available data for the analysis.

## Results

**Figure 1:**
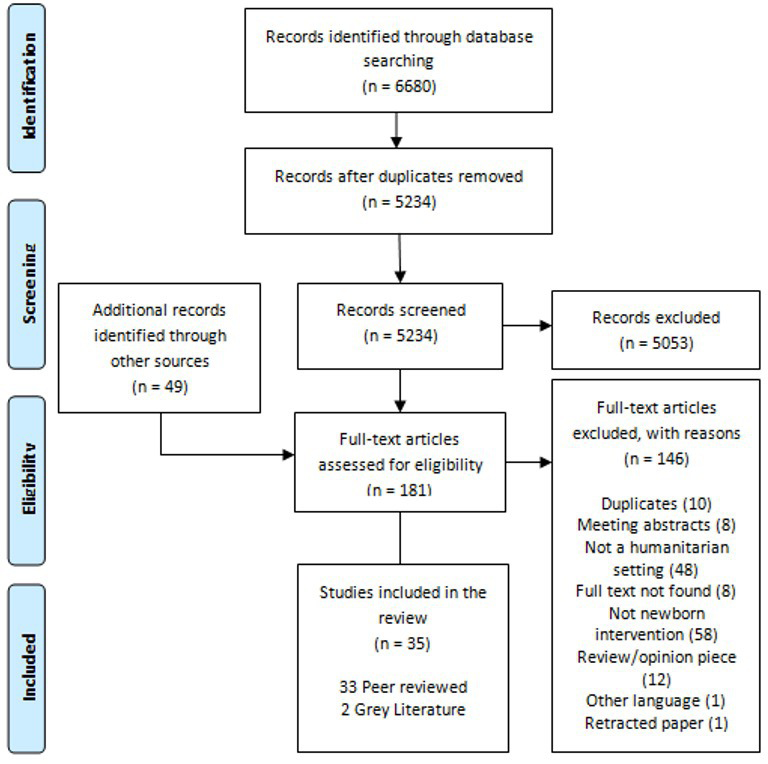
Preferred Reporting Items for Systematic Reviews and Meta-Analyses flow diagram.^19^

The databases search identified 5234 results. A total of 181 articles were screened full text; of which a total of 35 articles are included in this review, that is, 33 peer-reviewed^28–60^ and 2 grey literature publications^61 62^ (figure 1).

From the 35 studies included in this review, 21 studies were published between 2016 and 2021, 10 studies were published between 2011 and 2015 and the remaining 4 were published before 2010, the oldest one being from 1995.

### Study design and quality

The 33 peer-reviewed articles and 2 grey literature publications comprise: 1 cluster randomised trial,^51^ 17 cross-sectional studies,^30 31 34–37 39–42 46 48–50 52 58 60^ 2 cohort studies,^44 54^ 5 pre-post studies,^32 33 53 55 59^ 3 controlled pre-post studies,^29 47 57^ 2 case studies^43 45^ and 5 qualitative studies.^28 38 56 61 62^ Of these, 10 studies used a mixed methods approach^34 35 37 39 41 43 50 53–55^ (table 3).

**Table 3:** Characteristics of included publications

Study	Country	Crisis type	Population	Study design	Intervention	Key findings	Quality
**Peer-reviewed literature**							
Amsalu *et al*^36^	Somalia	Protracted conflict	General and internally displaced people (IDP)	Cross-sectional	Essential newborn care (ENC) at four primary healthcare (PHC) centres. Thermal care, feeding support, hygiene, newborn resuscitation.	Significant variation between health facilities (p<0.001) in the practice of skin-to-skin, support in breastfeeding initiation, early breastfeeding initiation and dry cord care.	High
Amsalu *et al*^55^	Somalia	Protracted conflict	General and IDP	Pre-post study	Eight-day training of healthcare workers (HCWs) at four PHC centres in ENC, including care of small babies; provision of supplies and medications and creation of a newborn health record system (following Field Guide recommendations). Five-day refresher training at 6 months.	Knowledge improvement, mean difference in score of +11.9% (95% CI 7.2 to 16.6) and from post-training to 18-month follow-up +10.9% (95% CI 4.7 to 17.0). Both with p<0.001. Improvement in ENC practices from baseline to endline with a difference in proportion of newborns that receive two or three ENC of +74.8% (95% CI 69.1 to 80.5) and +60.6% (95% CI 54.6 to 66.5) of newborns that received three ENC. Both p<0.001.	High
Amsalu *et al*^33^	Niger, Cameroon and Chad	Protracted crisis	Refugee	Pre-post study	Help Babies Survive training for health workers in PHC and hospitals (includes ENC and care of small babies) using a low-dose high frequency competence-building approach.	No change in neonatal mortality rate (NMR) - low at baseline and potentially under-reported - but positive trend. Health facilities improved and sustained the availability of medications, supplies and newborn clinical guidelines.	Low
Casey *et al*^37^	Burkina Faso, DRC and South Sudan	Protracted conflict	General, refugee and IDP	Cross-sectional	Elements of ENC (newborn resuscitation, breastfeeding support, newborn infection, thermal care, cord care).	Availability and quality varied across settings. In the DRC, none of the 25 health centres provide ENC; in South Sudan, 25% of health centres and none of the hospitals provided ENC and in Burkina Faso, 66.7% of hospitals, 25% of camp health centres and 9.5% of non-camp health centres provided ENC.	Medium
Chaudhary *et al*^45^	Nepal	Natural disaster	General and IDP	Case study	Implementation of Minimum Initial Service Package (MISP).	Deployment of 33 skilled birth attendants (SBAs) to birth centres; 1100 clean delivery kits and 53 000 newborn suits were provided across the 14 districts.	High
de Vries I *et al*^34^	Palestine	Protracted crisis	General	Cross-sectional	Postnatal home visits programme for high-risk cases and primiparous. Midwives and nurses were trained on several topics (including postnatal and newborn care), received postnatal home visit kits and had to conduct home visits within 48–72 hours post delivery.	Trained staff felt more confident about their knowledge and skills to assist women with breast feeding. Women that received post natal home visits demonstrated improved breastfeeding practices and behaviour changes that reduced harmful traditional practices.	Low
Dörnemann *et al*^58^	Afghanistan, Pakistan, CAR, DRC, Burundi, Sierra Leone	Protracted conflict (2), active armed conflict (1) and postconflict (3)	General	Cross-sectional	Médecins Sans Frontières (MSF) Operational Centre Brussels implemented low-tech specialised neonatal care units (SNCUs) in low-resource settings. The MSF SNCU model was characterised by an absence of high-tech equipment and an emphasis on dedicated nursing and medical care. Focus was on the management of hypothermia, hypoglycaemia, feeding support and early identification/treatment of infection.	Overall, 11 970 neonates were admitted, 41% of whom had low birth weight (<2500 g). The main diagnoses were low birth weight, asphyxia and neonatal infections. Overall, mortality was 17%, with consistency across the sites. Chances of survival increased with higher birth weight.	High
Draiko *et al*^57^	South Sudan	Protracted conflict	General	Controlled pre-post study	Helping Babies to Breathe training to hospital HCWs.	Knowledge and skills improved after training and declined 1-year post-training. Knowledge score mean difference of 55.2 (95% CI 50.9 to 59.6) and −15.0 (95% CI −22.7 to 7.4) a year after. P<0.001 for both. Mortality due to asphyxia decreased from 30.7% pre-training to 17.9% 1 year post-training (p=0.001).	Medium
Edmond *et al*^29^	Afghanistan	Protracted conflict	General	Controlled pre-post study	Conditional cash transfers to improve the use of health facilities and consequent postnatal care (PNC) visits. Financial incentives given to community health workers (CHWs) that visited pregnant women and referred them to a health facility for delivery; to women that delivered in a health facility and education/communication about the programme in the community.	Having at least one PNC visit increased in the intervention villages by 23.2%. Adjusted mean difference with control villages was 31.8%, 95% CI −0.05 to −0.68, p=0.080.	High
Eze *et al*^44^	Yemen	Protracted conflict	General	Cohort retrospective study	Outcomes of a neonatal intensive care unit with capacity for treatment of infection and jaundice, extra feeding support and oxygen therapy.	Most common diagnosis in this unit was prematurity. Most neonatal deaths were preterm newborns (90.1%) and 83.1% travelled for more than an hour to reach the facility. From the analysed predictors of facility neonatal mortality, travelling for >60 min to arrive at the NICU had an aOR=2.32 (95% CI 1.07 to 5.04, p=0.033).	High
Gee *et al*^28^	Kenya	Protracted conflict	Refugee	Qualitative	Social beliefs and practices of refugee populations around newborn care.	Skin-to-skin generally not practised and mothers are reluctant to practice. Women increasingly accept feeding colostrums, but mixed feeding practices are still present. Application of foreign substances to the umbilical cord is still practised.	High
Hoogenboom *et al*^46^	Thailand	Protracted conflict	Refugee	Cross-sectional	Elements of ENC at a PHC centre. Undefined immediate care, clean cord care and monitoring of newborn.	Appropriate immediate care (not specified) and cord care done in all observations and monitoring in the first hours occurred in 30% of the observations.	Low
Hynes *et al*^47^	DRC	Protracted conflict	General	Controlled pre-post study	Nine-month health workers training using participatory quality improvement (QI) approach comparing with workers that received only the standard 12-day training on ENC. ENC elements evaluated: clean cord care, antibiotic to eyes and weight.	Greater rate of change in ENC practices in group that received QI training (OR 49.62, 95% CI 2.79 to 888.28, p<0.05) and reached 100% ENC completion at endline.	Medium
Iellamo *et al*^35^	Palestine	Protracted crisis	General	Cross-sectional	Elements of ENC related to feeding, being practised by vulnerable women in the Gaza Strip.	63% of surveyed mothers practised early initiation of breast feeding and 42% reported given liquids other than breast milk to their newborn in the first 3 days of life. 18% of women reported receiving information about breast feeding during the contact with healthcare professionals.	Medium
Kim *et al*^30^	Afghanistan	Protracted conflict	General	Cross-sectional	Neonatal resuscitation in health facilities.	91% of the facilities performed neonatal resuscitation in the past 3 months. In the ones that did not, lack of training was the main reason.	Medium
Komakech *et al*^48^	Uganda	Protracted conflict	Refugee	Cross-sectional	Elements of ENC in refugee settlements. Initiation of breast feeding, cord care and skin-to-skin.	57% of mother breast fed in the first hour; 50.1% cleaned umbilical cord; 17.6% received skin-to-skin and 12.7% delayed bath. ENC was not being used and mainly not accessible to refugee mothers.	High
Krause *et al*^39^	Jordan	Armed conflict	Refugee	Cross-sectional	Implementation of the MISP.	Newborn resuscitation and advanced care was provided (no details given); clean delivery kits were provided to SBAs and pregnant women.	Medium
Kurdi *et al*^51^	Yemen	Protracted conflict	General	Cluster randomised trial	Monthly cash transfers, conditional of attendance to a monthly nutritional training session during 18–21 months.	Programme increased the probability of early breastfeeding initiation by 15.6% points (p<0.05) and of correct answers on breastfeeding initiations by 17.7% points (p<0.01).	High
Lam *et al*^49^	Global and country/region specific	Humanitarian settings (general)	General—INGOs	Cross-sectional	Survey of ENC reported by international and national non-governmental organisations, governments and UN Agencies.	Of the respondents, 62.5% reported having policies on maternal health and few (no data) reported having policies to address newborn care. Of the 27 surveyed organisations, only 36.7% reported proving newborn health training to their staff. And 51% lack of trained staff. ENC elements reported varied from 30% to 90%, highest being immediate drying, wrapping (80.4%), skin-to-skin contact and immediate breast feeding (87.5%) and least 48.2% promotion of disinfectants for umbilical cord care.	Medium
Lawry *et al*^50^	South Sudan	Protracted conflict	General	Cross-sectional	Survey of ENC elements (feeding, PNC and danger signs) and barriers to access services.	98.2% initiated breast feeding immediately; 7.9% (95% CI 6.1 to 9.8) of women received PNC 2 days after birth; 42.9% (95% CI 34.8 to 51) and 45.8% (95% CI 42.5 to 49.2) of men and women, respectively, could identify newborn danger signs.	High
Marsh *et al*^38^	Pakistan	Protracted conflict	General and refugee	Qualitative	Identify uncommon practices linked to a good newborn practice to mobilise communities for behaviour change.	Feeding, hygiene and thermal care practices were weak. Identified positive deviance practices on ENC and care of small and sick newborns. Communities were committed to behaviour change and to create support groups.	Medium
Massad *et al*^40^	Palestine	Protracted crisis	General	Cross-sectional	Assess the availability of neonatal units and its resources in Palestine.	Between Gaza, West Bank and East Jerusalem, 79% of neonatal units are in the West Bank. There is a shortage of equipments, medications and specialised human resources. There is a lack of referrals guidelines and challenges to do them on time.	High
McPherson *et al*^32^	Nepal	Armed conflict	General	Pre-post study	CHWs promoted desired behaviours related to maternal and newborn care through individual and group counselling.	ENC increased 19%–29%; nothing on cord, wrapped immediately and delaying bath p=0.000; wiped immediately p=0.001; breast feeding in first hour p=0.06 and attendance to PNC within 1 week of delivery p=0.01.	High
Miller *et al*^52^	Pakistan	Protracted conflict	Refugee	Cross-sectional	Training traditional birth attendants (TBAs) to improve the childbirth care. Included some elements of ENC (hygiene, cord care and breast feeding).	Compared with untrained TBAs, there was an increase in these practices.	Low
Mullany *et al*^59^	Myanmar	Protracted conflict	IDP	Pre-post study	Three-tiered network of community-based providers (TBAs, health workers and maternal health workers) that covered some elements of ENC (skin-to-skin, PNC and breast feeding in first hour).	Statistically significant increase in skin-to-skin prevalence rate ration (PRR)=2.70 (95% CI 1.93 to 3.78) and PNC PRR=2.07 (95% CI 1.81 to 2.37). Not significant change in breastfeeding initiation.	Medium
Myers *et al*^41^	Nepal	Natural disaster	General population	Cross-sectional	MISP implementation.	Availability of newborn care was low and equipment was malfunctioning or not available.	High
Newbrander *et al*^56^	Afghanistan	Protracted conflict	General	Qualitative	Traditional ENC practices in rural communities.	Newborns are usually immediately bathed; breast feeding is delayed until mothers clean their breasts; shame associated with seeking health services and inability for women to seek health services by themselves.	High
Zainullah *et al*^42^	Afghanistan	Protracted conflict	General	Cross-sectional	Knowledge and skills of health workers on neonatal resuscitation and care of small and sick neonates.	80.8% of doctors and 82.7% of midwives were trained in neonatal resuscitation. In some steps of EmONC decision-making skills, they scored low, that is, doctors scores 36.6% on essential actions when newborn does not cry. Knowledge about newborn infection signs/symptoms, treatment and care of LBW was below 60%.	Medium
Rosales *et al*^60^	South Sudan	Protracted conflict	General	Cross-sectional	Prevalence of ENC (breast feeding, thermal, cord and eye care) in rural community.	Breast feeding (74%) and wrapping immediately after birth (98%) 1% of newborns received all elements.	Medium
Sami *et al*^43^	South Sudan	Protracted conflict	IDP and refugee	Case study	Clinical training, supportive supervision and distribution of medical commodities in health facilities.	Difficulties in implementation were found in across the health system layers, that is, need for improvement of skilled workers, integration of newborn interventions in policies and in humanitarian funding scope.	Medium
Sami *et al*^31^	South Sudan	Protracted conflict	IDP	Cross-sectional	ENC and care of small babies in health facilities.	62.5% received thermal care; 74.8% infection prevention measures; 63.6% feeding support and 27.7% postnatal monitoring (weight and temperature taken).	High
Sami *et al*^53^	South Sudan	Protracted conflict	General	Pre-post study	Training of CHWs and facility health workers on ENC and care of small babies.	Mean knowledge scores among CHWs improved, 5.8 (SD=2.3) pre-training and 9.6 (SD=2.1) post-training; among facility-based health workers, 14.2 (SD=2.7) pre-training and 17.4 (SD=2.8) post-training. More confidence when caring for small newborns.	High
Turner *et al*^54^	Thailand	Protracted conflict	Refugee	Cohort	Implementation of a SNCU and staff training for the care of small and sick newborns.	Between 2008 and 2011, neonatal mortality in the camp declined by 51% from 21.8 to 10.7 deaths per 1000 live births (p=0.04) and mortality in premature newborns declined from 19.3% to 4.8% (p=0.03).	Low
Grey literature							
Krause *et al*^61^	Haiti	Natural disaster	IDP	Qualitative	Evaluation of the MISP.	Newborn care was available but varied across health facilities; access to services for sick newborns was a major concern. Recommendations to inform communities about where newborn care is available.	High
Krause *et al*^62^	Nepal	Natural disaster	General	Qualitative	Evaluation of the MISP.	Newborn care (not specified) was available varying between health facilities, lack of functioning equipment reported.	Medium

aOR, adjusted OR; CAR, Central African Republic; DRC, Democratic Republic of the Congo; INGO, international non-governmental organisation; NICU, neonatal intensive care unit; NMR, neonatal mortality rate.

Overall, 48.6% (n=17) of the studies were considered high-quality, 37.1% (n=13) medium-quality and 14.3% (n=5) low-quality (table 3). Most observational studies were medium-quality^30 35 37 39 42 49 60^ and high-quality^31 36 40 41 44 48 50 58^ despite the majority not including statistical methods to control for confounding and potential source of bias, although they do acknowledge these limitations. The controlled pre-post studies were considered medium-quality^47 57^ and high-quality,^29^ despite two not accounting for confounders in the design or analysis. The qualitative studies were medium-quality^38 62^ and high-quality^28 56 61^ despite only one considering their own role in creating potential bias.

### Study setting and main focus

The studies included in this review report evidence from 21 LMICs. Most interventions were implemented in sub-Saharan African countries (n=12), three in the Middle East, five in Asia and one in the Caribbean. Most of the evidence is from conflict-affected areas (n=27), three in postconflict settings, four in protracted crisis and four studies from areas affected by natural disasters (table 3).

The studies referred to different levels of care, with 16 studies referring to care at the community level, 14 studies referring to care at primary healthcare centres (PHCCs) and 11 studies referring to care in hospitals. Most included studies specifically focused on newborn health (n=15), nine focused on the continuum of care between mother and newborn and the remaining studies (n=11) focused on general SRH, maternal care or nutrition and included some newborn care interventions.

### Essential newborn care

#### Initiation of breathing

Neonatal resuscitation was the only specifically reported intervention on this topic across PHCCs and hospitals (n=9).^30 33 36 37 40 42 53 55 57^ Several studies assessed the readiness to perform neonatal resuscitation, the frequency of this intervention and the level of knowledge, skills and training of health professionals. Overall, these studies show that the provision of neonatal resuscitation was lacking in most of the assessed health facilities. Reported reasons were the lack of equipment (eg, term and preterm resuscitations bag and mask)^33 36 37^ and a gap in knowledge, specific training and decision-making skills.^30 33 40 42^

Training programmes carried out in conflict-affected areas in Somalia^55^ and in South Sudan,^53 57^ included stimulation and newborn resuscitation in their curriculums, and two of them specifically mentioned the Helping Babies to Breathe training programme.^63^ Overall, there was an increase in knowledge, skills and confidence to do bag and mask resuscitation without assistance.^53 55^

#### Thermal care

Eleven studies^28 31 32 36 48 49 53 55 56 59 60^ report specifically on thermal care practices across the different levels of care. Drying and warming the baby by wrapping were the most common and accepted interventions, practised almost universally among the reported settings.^31 32 36 49 60^

Skin-to-skin contact was not generally reported as being practised, and when practised, its frequency varied across levels of care.^28 31 36 48^ Studies from African countries refer to the resistance of mothers to accept this practice^28^ and lack of awareness and training of the health workers (HWs)^55^ as reasons for not practising it. Evidence from South Sudan shows that training of HWs increased the intention to promote skin-to-skin care,^53^ and in Myanmar this practice increased (10.1%–27.2%) with the creation of a network of trained community-based providers.^59^

Delaying bathing was specifically mentioned in five studies.^32 36 48 49 56^ This practice varied across settings, for example, in Somalia, it was observed in 99.2% (95% CI 97.1 to 99.9) of newborns born in the PHCCs,^36^ while in Uganda and Afghanistan it was not a common practice.^48 56^

#### Infection prevention and hygiene

Ten studies reported on hygiene practices across different levels of care.^28 31 34 36 46 47 49 52 55 60^ Hand washing was reported in five studies and its level of practice varied across settings.^36 46 49 52 55^ In a survey of humanitarian actors, 80% report promoting hand washing and use of clean delivery kits in their projects.^49^ In one setting, training of HWs improved general hygiene care by 24.1% (95% CI 18.4 to 29.7), although hand washing was still suboptimal.^55^

Provision of eye care with tetracycline was assessed at the health facility level in four studies and the results varied.^31 36 47 60^ In Somalia, 42.1% (95% CI 35.9 to 48.6) of newborns were observed to receive eye care^36^ while in South Sudan, one study reports that 6% of newborns received this care^60^ and in another nearly all (95.4%) of them received it.^31^

Umbilical cord care in the different communities was generally suboptimal, with social beliefs influencing the type of substance applied on the cord and still being common to apply foreign substances.^28 34 48 60^ In two settings, training seemed to improve the practice of dry cord care.^36 52^

#### Feeding support

Fourteen studies reported on early breastfeeding initiation (EBI).^31 32 35 48–53 55 56 59 60^ Four studies present high percentages of EBI being practised among women in the assessed hospitals, PHCCs and communities^31 50 59 60^; and one from a community in Uganda reports a lower percentage.^48^ At the broader level, 87.5% of surveyed humanitarian actors report that they promote EBI in their programmes.^49^

Several interventions seem to be able to enhance this practice. Two studies refer to trainings,^52 55^ for example, following the one done in PHCCs in Somalia, EBI increased by 53.6% (95% CI 46.4 to 60.9, p<0.001)^55^; in Nepal, following community messages this practice increased by 19%^32^ and in Yemen, a cash programme with nutritional training sessions increased the probability of EBI (p<0.05), although the sample size was small.^51^

Studies with a qualitative component revealed the social norms influencing breastfeeding practices. Among Somali refugee women, breast feeding was practised and it was accepted to give colostrum to the baby, although mixed feeding was still practised.^28^ Similar in the Gaza strip, where 42% of surveyed women confirmed that their newborns received liquids (other than breast milk) in the first 3 days of life.^35^ In Afghanistan, women did not initiate breast feeding immediately because they understood that they needed to first wash their breast.^56^

#### Monitoring of danger signs

Eight studies assessed the frequency of monitoring and knowledge about newborn danger signs from mothers, CHWs and facility HWs.^31 32 36 46 47 50 53 55^ In South Sudan, maternal knowledge was generally poor.^31 50^ From the settings where frequency of monitoring is reported, at PHCCs in Thailand, monitoring in the first hours of life was not a common procedure,^46^ and in South Sudan only 27.7% of newborns received postnatal monitoring.^31^

Following training of CHWs and HWs, in South Sudan there was an increase in correct responses by these professionals regarding the identification of newborn danger signs,^53^ while in Somalia the number of women who received this predischarge education was still suboptimal.^55^

#### Postnatal care checks

Eight studies reported about newborn postnatal care (PNC) visits either in the first 24 hours, day 3, between days 7 and 14 or all of the three visits.^28 29 32 34 36 49 50 59^ In Somalia, a study reported that 71% of women that delivered at the assessed PHCCs were followed up at home 7–9 days after discharge.^36^ The situation in South Sudan differed, where <10% of women received this care^50^; 83.3% of surveyed humanitarian actors reported providing PNC services, and of these, 65.2% were home visits for newborns within 3 days of birth.^49^ A postnatal home visits programme implemented in the Gaza strip showed that women included in the programme had improved breastfeeding practices, and it helped to reduce harmful traditional practices such as the application of oil and salt to the umbilical cord.^34^

A qualitative study reported on the reasons why Somali refugee women in Kenya would not seek routine PNC consultations and mentions the inconvenience to leave the house in the early postnatal period and long waiting lines as the main reasons for not attending postnatal health checks.^28^

#### Delaying cord clamping and administration of vitamin K

Two studies referred to the delay of cord clamping.^31 53^ In the assessed facilities in South Sudan, this practice was observed in 98% (95% CI 94.4 to 99.6) of hospital births and in 94.5% (95% CI 90.2 to 97.3) of PHCCs births.^31^ Qualitative data of the pre-post assessment reveals that midwives changed perspectives about delaying cord clamping and came to understand the reasons for it.^53^

Regarding the administration of vitamin K, three studies specifically included this practice.^31 36 55^ A cross-sectional study assessed five health facilities in South Sudan and reports that vitamin K was not available.^31^ In PHCCs in Somalia, 4.1% (95% CI 2.0 to 7.4) of newborns received vitamin K^36^ and after training and supplies provision it increased to 60%.^55^

### Care of small and sick newborns

Ten studies mentioned interventions that aimed at improving the care of small and sick newborns.^31 40 42–44 49 53–55 58^ A cross-sectional study from Afghanistan reports that knowledge on newborn infection signs/symptoms, treatment and care of low birthweight (LBW) newborns was one of the lowest scored topics.^42^ In South Sudan, at the hospital and PHCC level, it was observed that LBW newborns were not placed in Kangaroo Mother Care (KMC) and that there was a lack of equipment and materials to care for these neonates.^31^ At a broader level, 60% of humanitarian actors report providing KMC to preterm babies in their projects.^49^

Two pre-post studies^53 55^ referred to the implementation of trainings at the community and facility levels, which included topics to improve the care of small and sick newborns. At PHCCs in Somalia, there was a 40% improvement in knowledge and skills to care for LBW newborns with complications and HWs changed perspective on KMC.^55^ In South Sudan, CHWs knowledge to detect and refer sick newborns improved and facility HWs gained more confidence to care for small newborns.^53^ Another study found some challenges in the implementation of this intervention package, as staff did not have enough time to provide this specialised level of care and the hierarchy between HWs hindered treatment provision.^43^

Two articles describe the implementation of a specialised neonatal care unit with limited resources, showing that it is possible to implement such specialised care in crisis settings with outcomes within acceptable limits.^54 58^ Two other publications focus on neonatal intensive care units.^40 44^ In Yemen, this unit was prepared to provide all interventions, although there was no mention of the type of extra thermal care methods used.^44^

### Strategies to increase utilisation and effectiveness of newborn services

#### Community health interventions

Three studies^32 34 59^ report on the use of maternal-newborn community interventions programmes that increased the use of newborn care interventions. In Nepal, a birth preparedness package delivered through CHWs showed significant improvements in three ENC practices (p=0.00), PNC attendance within 1 week of delivery (p=0.02) but not in the use of skilled birth attendant.^32^ In Myanmar and Gaza, programmes with community-based providers increased the number of PNC visits and report improvement in some newborn interventions such as skin-to-skin and breastfeeding practices (statistically significant findings in Myanmar).^34 59^

A qualitative study in Pakistan with Afghani refugees and host populations conducted several interviews to understand the common newborn care practices and identify ‘positive deviant behaviour’ in these communities. These positive deviance findings were shared but there was no assessment of changed behaviour following this exercise.^38^

#### Training of healthcare workers

Six pre-post studies were done to understand the differences in knowledge and skills following training to CHWs and facility-based HWs, accounting for 40% of the studies reporting on strategies to increase utilisation of newborn health interventions.^33 47 52 53 55 57^ Overall, these studies note an improvement in newborn care knowledge and skills post-training, although the specific interventions assessed varied across studies.^47 52 53 55^ A low-dose high-frequency competency building approach was implemented in Niger, Chad and Cameroon where key health professionals were initially trained, to be able to begin a cascade of trainings in their own health facility, although no results were published on the impact of this intervention.^33^ A post-training assessment in South Sudan showed a significant decrease (p<0.001) in knowledge and skills over time and recommends the implementation of periodic refresher trainings to maintain the levels previously obtained.^57^

#### Provision of supplies, equipment and newborn data collection

Three studies specifically mention the provision of supplies and equipment as part of the implementation of ENC package as per the Field Guide.^33 43 55^ The availability of essential commodities improved, although equipment installation, poor supply management and stock outs of certain items, limited the sustained delivery of adequate care.^43 55^ One publication noted challenges with the use of a newborn registration book as it increased the workload of HWs.^55^

#### Financial incentives

Two studies involved the use of financial incentives. A cluster randomised controlled trial (RCT) in Yemen, of a cash transfer programme to women, conditional on their attendance to nutrition education sessions, evaluated the effect on EBI knowledge and reported practice and, report a significant increase in both indicators (p<0.05 and p<0.01, respectively).^51^ A controlled pre-post study done in Afghanistan assessed giving financial incentives to women who delivered at a health facility and to CHWs who referred pregnant women. While not being newborn focused, this programme increased PNC visits, although not statistically significant.^29^

### Use of the MISP and the Field Guide

Three studies assessed the effectiveness and feasibility of the Field Guide.^36 43 55^ They report an overall improvement in practices following implementation of the package (training of HWs, provision of medical commodities, equipment and installation of a newborn register). Two studies from South Sudan implemented part of the Field Guide, specifically the checklist of medical commodities^31^ and the training of CHW and facility HWs on newborn health interventions.^53^

Five publications refer to the implementation of the MISP,^39 41 45 61 62^ from which three are referring to the same humanitarian response following Nepal’s earthquake in 2015.^41 45 62^ None of the publications reported on the specific newborn care interventions.

### Implementors of newborn health interventions

Thirteen publications referred to activities carried out by or in partnership with ministries of health, but most of the included studies (n=22) were conducted in settings where non-governmental organisations (NGOs) were involved. The most common implementing international NGOs included International Medical Corps, MSF and Save the Children. The grey literature evaluations were done by WRC in partnership with CARE, Save the Children and International Planned Parenthood Federation.

## Discussion

This is the first review to assess the existing evidence on newborn health interventions in humanitarian settings, their effect on health outcomes and identify strategies used to increase their utilisation. There is a variation of the reporting of newborn practices across the studies. From the set of ENC interventions, thermal care and feeding support are the most commonly reported across the studies, while delaying of cord clamping and administration of vitamin K were the least. Regarding the care of small and sick newborns, details of the interventions used were mostly not mentioned, including on the use of KMC. The strategies used to increase utilisation of newborn care ranged from training of HWs, community-based interventions, financial incentives and provision of specialised equipment and supplies and their effect on health outcomes was not assessed.

There are some limitations to this systematic review. It may be the case that interventions implemented by local governments and smaller organisations are less likely to be documented, evaluated and published due to resource constraints. The lack of consistency of what is reported in each intervention group may not represent what is actually being implemented for newborns, therefore, not allowing for definite conclusions. Furthermore, given the heterogeneity of organisations delivering healthcare services in humanitarian settings (eg, national health systems, humanitarian agencies, etc) and, of models of aid delivery (eg, integrated health services vs vertical humanitarian programming), evidence is needed on the effectiveness of systems and deliveries for ENC and care of small and sick newborns. Despite most settings included being from conflict-affected regions, the contexts were highly varied which might limit the generalisability of these findings. Additionally, we were not able to cover all interventions that impact on newborn health on the continuum of care from preconception to postpartum in this review. While important, this broad lens was out our scope and because of this, this review might have missed publications with overlapping intrapartum interventions (eg, clean birth practices).

In most studies, ENC is reported as a general set of interventions but with a variation in the specific ones included. The five studies that use the Field Guide as a guideline reported more comprehensively on ENC compared with other studies. The last ones also had a larger maternal and child health focus, which might account for the under-reporting of specific aspects of newborn care as these were overlooked by more commonly reported maternal health interventions. The fact that EBI is a core tracker for ENC might also justify the fact that feeding support practices were more reported than other practices. The recent Lancet Series of 10 conflict settings case studies reports that newborn health, together with women and children’s health services, are not reported as being delivered in all case studies and only newborn care interventions included in the BEmONC and CEmONC are prioritised.^64^ The publications that focused on MISP were also lacking reporting details around newborn care. Therefore, it is not possible to draw on conclusions about the use of newborn interventions, as reporting differs across studies. This finding is consistent with a previous review of newborn care practices in sub-Saharan Africa which might point towards this being a general issue.^65^ Previous publications, although not specific for humanitarian settings, have called for the standardisation in reporting newborn care practices.^66 67^ Together with recent advances enlightening the situation of newborns, this could be a way to improve the available information on newborn care and consequently, improve the comparability of data, although it could also increase the burden on healthcare workers if not aligned with local and national information systems.

Training of HWs was the most common strategy to increase quality and use of newborn care practices. Most pre-post studies did not have a control group for comparison; they assessed the effect of trainings through post-training knowledge tests and observations, not including an analysis of newborn health outcomes.

Qualitative data provide fundamental information to promote the acceptance of these interventions, both by HWs and communities. For example, it allows for deeper understanding of local community practices and social beliefs,^28 56^ and the reasons behind the HWs not practising a certain intervention.^43^ This valuable information can assist to adapt programmes to the context and achieve the desired outcomes.

The included studies in this review were mostly high and medium quality but, because of the different designs, the use of different critical appraisal tools was necessary which made it difficult to compare the quality across the studies. In addition, the overall strength of evidence is low because of the lack of certain high-quality study designs, for example, experimental or quasi-experimental study designs which provide some statistical measure of difference between intervention and outcome, sufficient adjustment for potential confounders where appropriate and evidence of attribution. This finding is consistent with those from a recent systematic review on evidence of public health interventions in humanitarian settings, which reports that there is a gap in high-quality research on SRH topics.^15^ This is in spite of such research having been done in other health areas such as communicable diseases and mental health in crises settings—for example, by using a stepped wedge trial design which overcomes ethical issues raised in more traditional RCTs—which signals that it is possible to overcome the expected challenges of conducting rigorous research in these settings.^15^

There is also a gap in the diversity of settings and authors that specifically research newborn intervention using the Field Guide. The five studies that mention this guidance were done by only two authors in two countries. This could be explained by the fact that it has been published fairly recently (2018) and therefore postdates some of the papers identified in this review. It can also reflect challenges in implementing standardised approaches in settings with highly diverse health systems and on complex humanitarian emergencies, or a lack of awareness of this document.

Finally, acknowledging the continuum between maternal and newborn health,^68^ more research is needed to understand how intersectional inequities may affect the care of newborns in humanitarian settings.^69^ Such contextual knowledge is critical to designing and implementing impactful and locally appropriate programmes.

## Conclusion

Despite an increasing availability of guidance and advocacy on improving newborn health in humanitarian settings, there is still insufficient quantity and quality of studies that reflect this reality, by documenting these interventions and evaluating their effectiveness. Apart from the training of the HWs, community health interventions and understanding the social beliefs around newborn care seem to be key activities for the success of newborn care programmes. However, more research on these strategies, on systems and modes of delivery is still needed. The recent increase in publications focusing on newborn health will hopefully translate into further awareness and more consistent reporting of newborn practices by implementers, researchers and funders.

## Data Availability

All data relevant to the study are included in the article or uploaded as supplementary information.
