# Supplementary material for: A systematic review of newborn health interventions in humanitarian settings
Source: BMJ Glob Health. 2022 Jul 1;7(7):e009082. doi: 10.1136/bmjgh-2022-009082 (PMC9252185; doi:10.1136/bmjgh-2022-009082)
Supplement: Supplementary data [file bmjgh-2022-009082supp001.pdf]

Supplementary File 1: Search strategy for newborn health interventions in humanitarian crises settings

→ Protocol and tools available here:
https://discover.lshtm.ac.uk/discovery/fulldisplay?docid=alma991000841542503736&context=L&vid=44HYG_INST:44HYG_VU1&lang=en&search_scope=MyInstitution&adaptor=Local%20Search%20Engine&tab=Everything&query=any,contains,msc%20public%20health%20for%20development&facet=rtype,include,mscproject&facet=searchcreationdate,include,2020%7C,%7C2020&offset=0

SEARCH STRATEGY

Databases: Medline, Embase, Global Health and PsycINFO

1. LMICs Terms

296. Or/1-295 [ALL LOW AND MIDDLE-INCOME COUNTRIES] – Expert search for Medline and Embase

297. Developing Countries/
298. exp Asia/
299. exp Africa/
300. exp Europe, Eastern/
301. exp USSR/
302. exp Balkan Peninsula/
303. exp Caribbean region/
304. exp Central America/
305. exp Latin America/
306. exp South America/
307. exp Atlantic Islands/
308. exp Indian ocean islands/
309. Macau/
310. exp Pacific Islands/
311. Philippines/
312. Prince Edward Island/
313. Exp West Indies/
314. or/297-313
315. Japan/
316. 314 not 315 [Global Health and PsycInfo]

2. Humanitarian setting terms

317. Humanitarian.mp.
318. (disaster adj3 (relief or plan*)).mp.
319. ((relief or aid or rescue) adj2 work*).mp.
320. (typhoon* or hurricane* or cyclone*).mp.
321. (avalanche* or earthquake* or flood* or landslide* or tsunami*).mp.
322. Hurricane/

323. Avalanche/
324. Earthquake/
325. Flooding/
326. Landslide/
327. Tsunami/
328. Drought*.mp.
329. Drought/
330. exp natural disasters/
331. ((natural or victim) adj2 disaster).mp.
332. Disaster victims/
333. (starvation or famine*).mp.
334. Famine/
335. Starvation/
336. exp disasters/
337. exp Emergency medicine/
338. Disaster medicine/
339. Medical Missions/
340. ((armed or zone) adj2 conflict*).mp.
341. (conflict affected adj3 (population* or person* or communit*)).mp.
342. War*.mp.
343. exp Warfare/
344. exp Armed Conflicts/
345. exp war/
346. (refugee* or evacuee or evacuated).mp.
347. ((force* or population or human or internal*) adj2 displace*).mp.
348. (internally displaced adj2 (person or people)).mp.
349. Refugees/
350. Refugee Camps/
351. or/317-350

3. Newborn terms

352. (Newborn* or new-born*).ti,ab.
353. Neonate*.ti,ab.
354. Infant*.ti,ab.
355. Infancy.ti,ab.
356. Baby.ti,ab.
357. Babies.ti,ab.
358. exp Infant/
359. or/ 352-358

4. Interventions

360. “Minimum Initial Service Package”.ti,ab.
361. exp Perinatal care/
362. (antenat* or ante-nat* or prenat*).ti,ab.
363. Prenatal care/
364. (Postnat* or post-nat*).ti,ab.

365. Postnatal care/
366. ("Emergency newborn care" or "emergency new-born care" or "emergency neonat*").ti,ab.
367. (emnc or bemnc or cemnc).ti,ab.
368. ((Basic or comprehensive) ADJ2 (Emergency newborn care)).ti,ab.
369. ((neonatal or newborn or new-born) ADJ2 resuscitation).ti,ab.
370. ((neonatal or newborn or new-born or infant or baby) ADJ2 (health or care)).ti,ab.
371. Exp newborn care/
372. "Helping babies breath".ti,ab.
373. Childbirth*.ti,ab.
374. Birth/
375. Parturition/
376. (clean ADJ2 (deliver* or birth*)).ti,ab.
377. Delivery, Obstetric/
378. Skilled birth attend*.ti,ab.
379. ((Newborn or new-born) adj2 (danger sign*)).ti,ab.
380. "Essential newborn care".ti,ab.
381. (umbilical cord adj3 (care or hygiene)).ti,ab
382. (delay* adj2 cord clamping).ti,ab.
383. Thermal care.ti,ab.
384. Vitamin K Deficiency Bleeding.ti,ab.
385. Vitamin K Deficiency Bleeding/
386. ((Newborn or new-born or neonatal) ADJ2 infection*).ti,ab.
387. (Prematur* or preterm).ti,ab.
388. ("Low birth weight" or LBW).ti,ab.
389. Exp low birth weight/
390. (Small ADJ2 (baby or babies)).ti,ab.
391. skin-to-skin contact.ti,ab.
392. ("Kangaroo mother care" or KMC).ti,ab.
393. Kangaroo-Mother Care Method/
394. Breastfeed*.ti,ab.
395. Breast Feeding/
396. Infant health/
397. exp Infant Care
398. exp Infant, Newborn, diseases/
399. or/ 360-398

400. 296 and 351 and 359 and 399
401. Limit 400 to yr =1990-2021 (In Embase and Medline)
402. 316 and 351 and 359 and 399
403. Limit 402 to yr=1990-2021 (In Global Health and PsycINFO)
